# Taste Function in Adult Humans from Lean Condition to Stage II Obesity: Interactions with Biochemical Regulators, Dietary Habits, and Clinical Aspects

**DOI:** 10.3390/nu15051114

**Published:** 2023-02-23

**Authors:** Alessandro Micarelli, Alessandra Vezzoli, Sandro Malacrida, Beatrice Micarelli, Ilaria Misici, Valentina Carbini, Ilaria Iennaco, Sara Caputo, Simona Mrakic-Sposta, Marco Alessandrini

**Affiliations:** 1 Unit of Neuroscience, Rehabilitation and Sensory Organs, UNITER ONLUS, 02032 Rome, Italy; 2 Institute of Clinical Physiology, National Research Council (CNR), 20162 Milan, Italy; 3 Institute of Mountain Emergency Medicine, Eurac Research, 39100 Bolzano, Italy; 4 L-Nutra Italia S.r.l., 20122 Milan, Italy; 5 ENT Unit, Department of Clinical Sciences and Translational Medicine, University of Rome Tor Vergata, 00133 Rome, Italy

**Keywords:** obesity, taste, leptin, ghrelin, dietary habits

## Abstract

Differences in gustatory sensitivity, nutritional habits, circulating levels of modulators, anthropometric measures, and metabolic assays may be involved in overweight (OW) development. The present study aimed at evaluating the differences in these aspects between 39 OW (19 female; mean age = 53.51 ± 11.17), 18 stage I (11 female; mean age = 54.3 ± 13.1 years), and 20 II (10 female; mean age = 54.5 ± 11.9) obesity participants when compared with 60 lean subjects (LS; 29 female; mean age = 54.04 ± 10.27). Participants were evaluated based on taste function scores, nutritional habits, levels of modulators (leptin, insulin, ghrelin, and glucose), and bioelectrical impedance analysis measurements. Significant reductions in total and subtests taste scores were found between LS and stage I and II obesity participants. Significant reductions in total and all subtests taste scores were found between OW and stage II obesity participants. Together with the progressive increase in plasmatic leptin levels, insulin, and serum glucose, decrease in plasmatic ghrelin levels, and changes in anthropometric measures and nutritional habits along with body mass index, these data for the first time demonstrated that taste sensitivity, biochemical regulators, and food habits play a parallel, concurring role along the stages evolving to obesity.

## 1. Introduction

Since the sense of taste may impact nutrient selection and dietary intake [1], studies in the last decades have demonstrated an increasing interest in deepening the effect of the taste on energy balance, satiety, and long-term health [2,3]. Rising findings evidence that the gustatory system is also possibly involved in many other important metabolic processes such as energy homeostasis and appetite control, and in turn conditioning health and body weight [4,5]. Considering that sweet taste has an important hedonic appeal, the choice of sweet nutrients has been considered crucially involved in the control of weight and the development of obesity [6]. Given these aspects, obesity has been considered a global health concern of great magnitude [7], and efforts devoted to mitigating the epidemic have been almost unsuccessful [8]. Despite the underpinnings of obesity being various, key factors—together with unusual daily physical activity [9]—consist of overconsumption of cheap, highly palatable, energy-dense, and nutrient-poor foods and beverages with high concentrations of sugar [10]. 

In the field of sweet perception—for example—different psychophysical methods to estimate the sense of taste are used to depict different features of the tastant [11]. A widely used technique consists of threshold testing, which may determine either the lowest sweet tastant concentration of detection or recognition [12]. In this vision, those persons having a lower intensity perception of sweetness are more prone to obtaining higher quantities of a sweet tastant to be satisfied and their higher hedonic liking of sweetness at elevated concentrations may induce them to increase the usage of sweet foods [13]. 

Interestingly, if on one side the sense of taste contributes to obesity development, on the other one it seems to be itself also affected by obesity given that evidence suggests that the primary gustatory tissue, the tongue, is an obesity target organ [14]. Furthermore, different modulators such as leptin, ghrelin, and insulin and the plasma concentration of glucose have been demonstrated to be involved in the sweet sensation of sweet-sensing taste cells [15,16] (for example, a certain degree of reduction in taste bud percentage has been associated with obesity [14]) and to modulate gustatory pathways at higher levels [16,17]. 

However, the literature did not fully elucidate if differences in taste sensitivity might reflect the different stages of obesity and their relationship with dietary habits, anthropometric aspects, circulating levels of modulators of feeding and taste behavior, and metabolic assays.

Thus, since obesity stages have been demonstrated to be related—in terms of body mass index (BMI)—with different risks of metabolic diseases, mortality, and survival [18,19] and to be involved in nutritional choices [20], the aim of the present study was (i) to evaluate the changes in gustatory sensitivity, dietary habits, circulating levels of biochemical regulators of feeding and taste behavior, anthropometric aspects, and metabolic assays between healthy lean subjects (LS) and participants affected by overweight (OW) and stage I and II obesity and (ii) to estimate the possible impact of these factors on total taste score when evaluating all the participants together.

## 2. Materials and Methods

### 2.1. Participants

The sample consisted of four groups: 60 LS (29 female), 39 OW participants (19 female), 18 participants with stage I obesity (11 female), and 20 participants with stage II obesity (10 female). All the participants were Caucasian adults and were recruited from the University Hospital of Rome “Tor Vergata”. All subjects underwent a general clinical and ear-nose-throat (ENT) examination. Current or recent smokers (<3 years of abstinence) and individuals affected by allergies, metabolic diseases, and a history of ENT surgery were excluded. Individuals with endocrinological disorders or suffering from chronic renal disease and other systemic or organ failure disorders including neuropsychiatric and cardiovascular disorders, as evaluated by medical history, physical examination, and routine blood tests were further excluded. Conditions of vegetarian/vegan diet, history of gustatory disturbances, ongoing use of medication (except oral contraceptives), and drug/alcohol abuse were considered as exclusion criteria. Gastrointestinal/eating disturbances and surgery were considered dropout conditions. Pregnant and currently breastfeeding females were excluded [21,22]. After inclusion, participants provided written informed consent. The study was performed in agreement with the Declaration of Helsinki and was approved by the Institutional Ethics Committee (Reference number RS 60/20, date of vote: 24 July 2020). Following other research and clinical experiences all the participants filled out a food frequency questionnaire (FFQ) defined according to local dietary habits [23]. The final report recorded the intakes of calories (energy intake; EI), fats (saturated, monounsaturated, unsaturated), carbohydrates, proteins, fibers, and relevant micronutrients [23]. Thus, following the Goldberg cut-off method, which was developed to identify individuals whose reported energy intake would be considered a plausible habitual intake and to exclude participants who report intakes that are unlikely to represent true habitual intake [24], the ratio of EI:basal metabolic rate (BMR) for each participant was calculated by dividing self-reported FFQ energy intake by BMR estimated by means of the equations of Schofield [25]. Therefore, subjects with calculated values of the ratio EI: BMR in the interval 1.10–2.19 were classified as “plausible or adequate energy reporters”. Subjects with individual EI: BMR < 1.10 and/or individual EI: BMR > 2.19 were categorized as implausible reporters [26].

### 2.2. All the Participants Underwent

#### 2.2.1. Anthropometric Parameters and Bioelectrical Impedance Analysis (BIA) Measurements

In line with other experiences, height and waist circumference (WC) were respectively measured to the nearest 0.01 m using a Stadiometer (Holtain Ltd., Crymych, UK) [27] and in cm [28]. Following previous experiences and BIA devices (Omron HBF-500 BIA, Omron Medizintechnik, Mannheim, Germany) [29,30], weight was measured in kilograms (Kg) and fat mass (FM, in %), skeletal muscle mass (MM, in %), grade of visceral fat (VF level), and resting energy expenditure (REE, in kilocalories (Kcal)) were calculated by means of the manufacturers’ equations [31,32,33].

#### 2.2.2. Taste Function Testing

Participants’ taste functions were tested between 7.00 to 9.30 a.m. after abstaining from food or non-water beverage since 10 p.m. of the previous night [34]. One hour prior to testing, all the participants did not eat or drink anything except water and they did not brush their teeth. The taste test—consisting of filter paper strips (“Taste Strips”, Burghart, Wedel, Germany) impregnated with four concentrations of the four basic taste qualities: sweet, sour, salty, and bitter (for details see [35])—was administered in increasing concentrations (0.4, 0.2, 0.1, and 0.05 g/mL sucrose; sour: 0.3, 0.165, 0.09, and 0.05 g/mL citric acid; salty: 0.25, 0.1, 0.04, and 0.016 g/mL sodium chloride; bitter: 0.006, 0.0024, 0.0009, and 0.0004 g/mL quinine hydrochloride) and by means of a randomized method on the left or right side of the anterior third of the extended tongue, resulting in a total of 32 trials. Before each administration of a strip, the mouth was rinsed with water. With their tongue still extended, participants were asked—by means of a multiple forced choice method—to identify the taste from a list of the four qualities. After the number of correctly identified tastes per side was summed, the left and right sides scores were added up in order to obtain the total number of identified tastants [35]. The procedure lasted about 20 min for lateralized testing [36]. 

#### 2.2.3. Biochemical Assays 

After a 12 h fast, baseline laboratory parameters, including serum glucose, creatinine, alanine aminotransferase (ALT), aspartate aminotransferase (AST), total cholesterol, triglycerides (TGs), high-density lipoprotein (HDL) cholesterol, and low-density lipoprotein (LDL) cholesterol, were measured using the auto analyzer system (Aeroset System Abbott, Abbott Laboratories, Diagnostic Division, Chicago, IL, USA) [37]. 

Further, once plasma samples were collected following previous procedures described elsewhere [21], leptin levels were measured by means of an enzyme immunoassay (ELISA) kit (cat. No. EH0216; FineTest, Wuhan, China), insulin was analyzed by an immunoradiometric assay (BioSource International, Camarillo, CA, USA), and ghrelin was determined by an enzyme-linked immunosorbent assay kit (cat. No. EH0355; FineTest, Wuhan, China). The methods of analysis and concentration measurements followed established procedures [21]. 

#### 2.2.4. Data Handling and Statistical Analysis

In line with those works powerfully associating obesity alterations and BMI [17,38], the sample size was calculated to detect intergroup differences in the BMI results. The sample size for the test hypothesis was calculated in accordance with the context (independent samples and continuous variables), using a statistical power of 80% (1 − β) for an error probability of 0.05. The *t*-test for independent samples and an effect size of 0.80 was used. At least 12 participants per group were thus determined to be included and the sample size was finally in accordance with previous experiences [17,21,38]. The X^2^ test was performed to assess associations between categorical factors and groups. Descriptive data were computed as mean ± standard deviations (SDs) for FFQ scores, gustatory testing, biochemical assay parameters, and BIA measurements. To define that data for independent samples were of Gaussian distribution, D’Agostino K squared normality and Levene’s homoscedasticity tests were performed (under the null hypothesis that the data were normally and homogeneously distributed). A between-group analysis of variance was carried out for each of the FFQ values, taste testing scores, biochemical assay parameters, and anthropometric/BIA measurement variables. BMI degree and gender were treated as categorical predictors while age was treated, where possible, as a continuous predictor. The significant cut-off level (α) was set at a *p*-value of 0.05. Bonferroni correction for multiple comparisons was used for the post hoc test of the significant main effects, and the corrected level of significance was set at 0.008 (0.05/6). Given the exploratory nature of the study and previous biomedical retrospective approaches [39], the associations between the total taste score and three groups of prognostic factors (anthropometric, demographic and BIA variables, FFQ-based nutrient intakes, and biochemical assays) were examined by three different multiple regression analysis models. *p*-values less than 0.05 were considered statistically significant [40] (STATISTICA 7 package for Windows). 

## 3. Results

Socio-demographic aspects of the four groups of participants are reported in Table 1. Regarding anthropometric and BIA measurements, participants affected by stage II obesity had a significantly increased WC (*p* < 0.001), weight (*p* < 0.001), BMI (*p* < 0.001), VF level (*p* < 0.001), and REE (*p* = 0.0059) when compared to stage I obesity participants (Table 1). Both these groups had significant (*p* < 0.008) increases when compared with both LS and OW participants in WC, BMI, FM, VF level, REE, and reduction in MM. A similar significant behavior in these anthropometric and BIA measurements was found also found between LS and OW participants (Table 1).

Once we applied the Goldberg cut-off method and given the estimated BMR in the total subjects (1651.83 ± 270.64 Kcal in LS; 1858.97 ± 525.83 Kcal in OW; 2753.66 ± 1057.43 Kcal in stage I obesity; 2892.9 ± 833.05 Kcal in stage II obesity), out of the 137 participants, 58 were found as implausible FFQ reporters (29 LS; 13 OW; 7 stage I obesity; 9 stage II obesity). 

**Figure 1:**
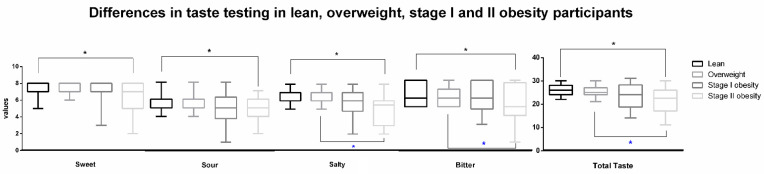
Main differences (mean ± standard deviation) between lean, overweight, stage I, and II obesity participants in taste testing. Significant differences are depicted with asterisks with different colors for different comparisons (black: lean–stage II obesity; blue: overweight–stage II obesity) and brackets.

Significant (*p* < 0.008) differences in terms of FFQ scores were found between the four groups of plausible reporters, depicting a progressive intake increase in nutrients along the BMI stages (Table 2), with no significant differences between stage I and stage II obesity participants. A significant reduction in total and all subtests taste scores between LS and stage II obesity participants (*p* < 0.001, *p* = 0.007, *p* = 0.002, *p* < 0.001, and *p* = 0.001, respectively) (Figure 1). Similarly, a significant reduction in total, salty, and bitter taste scores was found between OW and stage II participants (*p* < 0.001, *p* < 0.001, and *p* = 0.008, respectively). No significant differences in total (and subtests) taste scores were found between stage I and II obesity participants nor between lean and OW participants, between this latter group and stage I obesity participants, and between LS and stage I obesity (Figure 1). A significant interaction was found for the total taste and sour taste scores with factor “gender” (F(1, 135) = 8.18, *p* = 0.004; F(1, 135) = 8.76, *p* = 0.003), depicting that female participants outperformed male participants along these tests.

**Figure 2:**
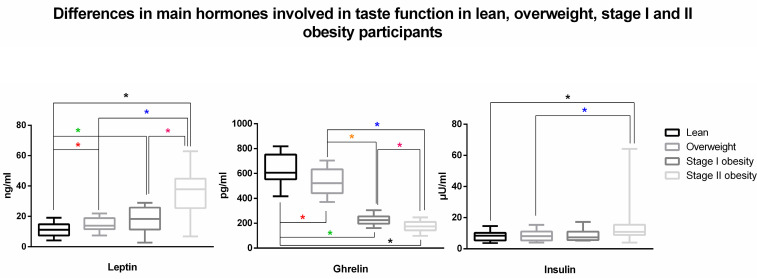
Main differences (mean ± standard deviation) between lean, overweight, stage I, and II obesity participants in hormonal profile involved in taste function. Significant differences are depicted with asterisks with different colors for different comparisons (black: lean–stage II obesity; green: lean–stage I obesity; red: lean–overweight; blue: overweight–stage II obesity; orange: overweight–stage I obesity; violet: stage I–stage II obesity) and brackets. Nanogram: ng; picogram: pg; milliliter: mL; micro international unit: µU.

Further, stage II obesity participants demonstrated significantly higher levels of plasmatic leptin, serum glucose, and lower levels of plasmatic ghrelin with respect to stage I obesity (*p* < 0.001, *p* = 0.001, and *p* < 0.001, respectively) and significantly higher levels of plasmatic leptin, insulin, and serum glucose and lower levels of plasmatic ghrelin with respect to OW (*p* < 0.001, *p* = 0.002, *p* < 0.001, and *p* < 0.001, respectively) and LS (*p* < 0.001, *p* < 0.001, *p* < 0.00,1 and *p* < 0.001, respectively). Stage I obesity participants had significantly higher levels of plasmatic leptin (*p* < 0.001) and serum glucose (*p* = 0.001) and lower levels of plasmatic ghrelin (*p* < 0.001) with regard to LS and lower levels of plasmatic ghrelin (*p* < 0.001) with respect to OW participants. The latter group was found to have significantly higher levels of plasmatic leptin (*p* < 0.001) and serum glucose (*p* = 0.001) and lower levels of plasmatic ghrelin (*p* < 0.001) with regard to LS (Table 3, Figure 2). Further significant differences in biochemical results depicting a worsening of metabolic pattern in the four sub-groups are depicted in Table 3.

**Figure 3:**
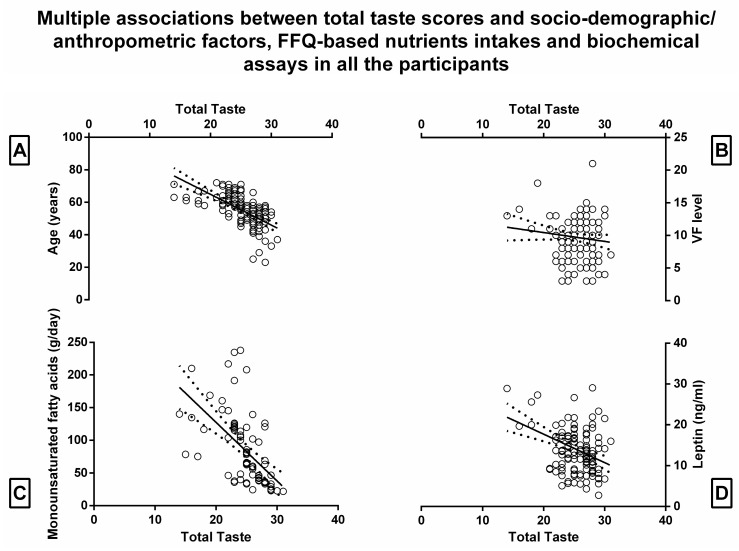
Plot depicting multiple associations in all the participants in the regression model between total taste function score and socio-demographic/anthropometric factors (**A**,**B**), food frequency questionnaire (FFQ)-based nutrient intakes (**C**), and biochemical assays (**D**). nanogram: ng; gram: g; visceral fat: VF; milliliter: mL.

When performing multiple regression analysis within all the participants in order to determine the total taste score in relation to the three prognostic factors (anthropometric, demographic and BIA variables, FFQ-based nutrients intakes, and biochemical assays) the multiple correlation coefficient was, respectively, 0.75, 0.54, and 0.45, with a *p*-value less than 10^−4^. The regression was statistically significant only for age, VF level, and gender with partial correlation coefficients of −0.45, −0.17, and −0.2, respectively; for monounsaturated fatty acids with a partial correlation coefficient of -0.20; for plasmatic leptin levels with a partial correlation coefficient of −0.36 (Table 4, Figure 3).

## 4. Discussion

The main interesting findings of the present study reside in the significantly decreased taste sensitivity when comparing stage II obesity participants to LS and OW. For the first time, all the taste sensitivities were found to be significantly reduced—as well as the total taste score—when comparing LS to the advanced stage of obesity, while the salty and bitter—and their sum sensitivities were significantly reduced when comparing OW and stage II obesity (Figure 1). On the other side, participants exhibited significant alterations in biochemical assays connected with both taste sensitivity and the worsening of metabolic status along with the BMI increase (Table 3, Figure 2). As expected, significant progressive increases in energy intake and in the amount of the main nutrients involved in obesity development were found (Table 2) [41]. 

All these aspects together tend to corroborate all previous studies evidencing that obesity is fostered by individual, nutritional, and sedentary lifestyle factors that may in turn provoke an excess of caloric intake [42]. Within individual factors, taste identification plays a pivotal role in food preferences, choices, consumption [43], and—as a consequence—body weight regulation. This evidence is supported by those studies linking taste to food selection and obesity [16,44] and recent reviews remarking that nutrient intakes or food habits may in turn impact taste sensitivity [1]. 

Although the association between BMI and taste perception has been studied extensively with heterogeneous results over the past decade, it has been repeatedly reported—in line with the results of the present study—that BMI increase is associated with a reduction in the perceived intensity of different tastants and weakened sense of taste [42,44,45,46,47]. In light of this, a reduction in salty sensitivity was found to be associated with higher BMI [47]. Other works supported this evidence, demonstrating that obese adults have a less intense sweet and salty taste perception [46] or that women affected by obesity perceive the monosodium glutamate detection threshold as increased (less sensitive) [45]. However, in the latter study, no differences in sucrose threshold were found between obese and lean participants [45]. Of note, the work of Hardikar et al. [44] contrasted such evidence when observing in obese participants a lower sucrose and sodium chloride threshold when compared to lean subjects. Similarly, Bartoshuk and coworkers [48] found that the perception of sweet and fatty tastes was increased in those subjects with higher BMI, concluding that individuals with obesity might have an increased sensitivity for these tastants. The inverse relationship between weight and taste sensitivity has been further confirmed by all those studies demonstrating increased sweet, salt, sour, and bitter sensitivity in patients undergoing bariatric surgery, both after Roux-en-Y gastric bypass and vertical sleeve gastrectomy [9,49]. Many of these studies concluded that post-surgical taste changes could have been exacerbated by weight loss, suggesting a causal relationship between weight and taste perception [49,50] and positing that reduced sweet taste threshold could be the consequence of the reduced intake of sweet and energy-dense foods [1,51,52]. In light of these data, some authors claimed that the greater the ability to perceive a certain taste, the lower the preference for it, resulting in a lower intake of that food [12,53]. However, although associations between regulation of body weight and alteration in taste seem to exist and may underpin the variety of responses to weight loss interventions, it cannot be excluded—also due to the large inter-individual taste changes differences—that other factors than weight loss per se, such as reward value and gut–brain interaction, might drive the observed changes in taste perception [1,16]. 

At hormonal levels—in line with the current literature—patients affected by OW and stages I–II of obesity demonstrated a progressive increase in leptin and insulin and a reduction in ghrelin (Figure 2), possibly impacting the imbalance of the taste signaling and food reward [54,55,56]. The latter hormone, indeed, a regulator produced in the stomach, has been found to have orexigenic effects at the peripheral and central levels [57]. If it has been demonstrated that ghrelin is able to control the response to food cues via a neural pathway involved in the regulation of feeding and, most importantly, in the appetitive response to food cues by increasing hedonic and incentive responses to adequately influence food intake [57,58,59], specific knock-out of ghrelin, growth hormone secretagogue receptor, and the ghrelin O-acetyltransferase (needed for the post-transcriptional activation of ghrelin) in mice resulted in altered responses to salty and sour food stimuli [60,61]. This resulted in lower consumption of sweet solutions (sucrose and maltodextrin), reduced weight gain, and improved glucose and insulin homeostasis [61]. On the other side, given the fact that (i) insulin was found to down-regulate taste buds’ cell expression in a dose-dependent manner [62] and that (ii) obesity is associated with the development of insulin resistance, alterations in the sense of taste have been speculated to be a consequence of metabolic disturbances related to extreme overweight [16]. Indeed, although genetic variations in taste receptors have been ascertained as driving genetic–environment interaction in the development of taste behavior and obesity [63,64], an increasing body of evidence demonstrated that different hormones may also modulate taste bud activity. In this vision, leptin acts via binding to its receptor obese receptor in type II taste buds cells and interferes with local K_ATP_ channels [15,65]. Thus, beyond one of the pivotal roles of leptin in energy homeostasis being to boost hunger in response to its decreased or absent circulating levels [66], it has been found that the activation of such channel results in reduced sweet response signaling to the afferent nerve fiber in the taste cell and dampens sweet perception [15]. Considering that leptin levels in the bloodstream may strongly correlate with BMI [16] and that leptin may mitigate sweet taste sensitivity, it may account for the reduction in sweet taste sensitivity often observed in people with obesity [48,67]. Anecdotally, such hormones are also present in saliva which is not a simple byproduct of the plasma and different studies demonstrated that the concentrations of these (and other) regulators are mainly due to transport from the blood vessels into the glandular cell [68]. However, recent studies also demonstrated that salivary gland production is present and may modulate taste responses in relation to physiological status [69]. 

Some of these aspects have also been confirmed in the present study by the regression model, which highlighted a negative association between total taste score and plasma leptin levels among all the subjects and—even if beyond the level of significance—the insulin plasma level, possibly due to the downregulation of several taste bud cell genes demonstrated in the literature [62] (Table 4, Figure 3, Table S1). Further, according to previous experiences [42], the regression models evidenced that other variables impacting taste sensitivity are age (negative association), gender (women generally show higher sensitivity), and VF level (negative association) (Table 4, Figure 3). These aspects tend to corroborate those previous experiences positing that age is associated with an overall reduction in the number of taste buds and the number of taste cells per taste bud, especially in men [42,70,71], and that such taste sensitivity reduction is the effect or the cause of the BMI increase, on which constitution visceral fat has a contributing effect [42]. This result is noteworthy since it supports previous findings in mice [14] showing that chronic low-grade inflammation brought on by obesity reduces the number of taste buds in gustatory tissues of mice and is likely to be the cause of taste dysfunction seen in obese populations [72]. In this vision, VFlevel—more than the other anthropometric measures that collectively depict obesity (i.e., BMI and FM%) and that have been found to be negatively (even if not significantly) associated with total taste score (see Table S1)—is more metabolically active than subcutaneous fat [73] and affects the development of metabolic disturbances by contributing to the pro-inflammatory milieu [74], possibly impacting taste bud decrease.

Further, together with the interaction found between total taste and sour taste score with the gender factor, the regression model confirms the general trend in the literature suggesting that women exhibit higher gustatory sensitivity than men [36,75,76,77]. Similar findings have been reported for the other chemical sense, olfaction, where women also outperform men [21,78,79,80]. Besides the fact that the exact reason for female superiority in olfaction and taste remains unexplained, one possible explanation relates to a hormonal influence and protective effect on the chemical senses [36,81,82,83]. However, in contrast to olfactory function, taste function seems to be more influenced by hormonal changes as shown during pregnancy [84].

Finally, with regard to relationships between total taste scores and FFQ-based nutrient intake, a negative association between monounsaturated fats and taste sensitivity was found, corroborating those theories evidencing that the consumption of enriched in sugar and fat diets is associated with a reduction in taste stimuli sensitivity, thus impacting food choices and fostering food intake [14,46,48,85,86]. At the same time, such a negative association reinforces those previous studies in which significant increases in fat perception were observed following a low-fat diet [87,88], indicating that differences in taste sensitivity to fatty acids may be a result of gustatory adaptation to a high-fat diet and may contribute to excess fat intake because of an attenuated taste response to fatty acids among individuals who habitually consume a high-fat diet, as happens in overweight/obese subjects [88,89]. These phenomena have been attributed to a downregulation in the expression of specific subunits of sensing G-protein coupled receptors of different nutrients, which in some cases, given their cross-sensitivity, may also account for a downward trend for the sensitivity regarding other tastants [90,91]. With the premise that specific tests targeted on different nutritional stimuli have not been used in the present study, the complexity of these phenomena could be one of the underpinnings of those (not significant) associations found in the present study (see Table S1), corroborating previous works which highlighted that one taste quality threshold might be affected not only by the deprivation of its stimulus but also by the exposure to another one [92,93,94]. 

**Figure 4:**
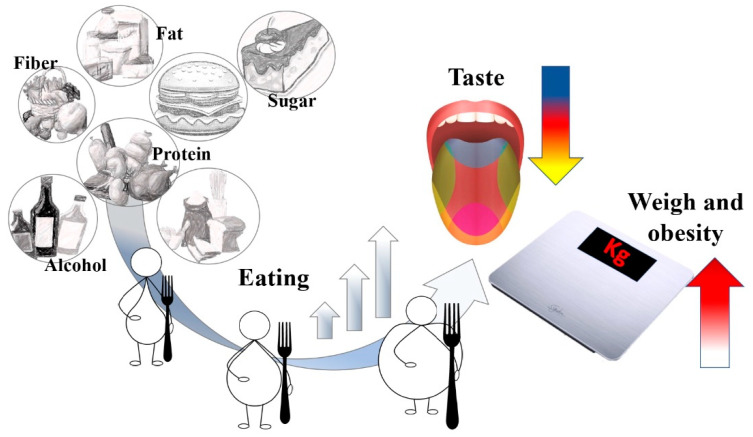
The consumption of high-fat foods, proteins, fibers, and sugars and/or alcohol abuse reshapes taste sensation, influencing and promoting the food intake, creating a vicious circle that, over time, leads to weight gain and increases the risk of diseases.

In conclusion, the present large-scale study for the first time demonstrated that—among individual factors—taste sensitivity, biochemical regulators, and food habits play a parallel, concurring role along the stages evolving to obesity (Figure 4). Considering that taste plays a pivotal role in the mosaic pathways influencing food preferences, choices, and thus, consumption [43,95] and that it has been found to be clearly associated with certain hormonal and anthropometric aspects and food habits, further studies deepening the role of taste in food choices and eating behavior are pivotal to enlarging the comprehension of the factors involved in body weight maintenance and the risk of chronic diseases including obesity, atherosclerosis, cancer, diabetes, liver disease, and hypertension.

## 5. Limitations of the Study

Although the current study addresses a number of methodological gaps in the literature by controlling important potential confounders, there are limitations to consider. First, the biochemical regulator assay considered in the study is restricted and it needs to be enlarged in future protocols to other new hormonal compounds and targets, such as those that could partially depict the association between gustatory behavior and the brain reward system. Secondly, the authors are aware that sensory processing involved in overeating and obesity development is a complex mosaic of underpinning factors including individual genetic variations, cultural and psychological factors, and other chemosensory pathways, and that taste “per se” could not explain the whole process [96]. In light of this, despite the present study sample being one of the largest in which the gustatory sensitivity has been analyzed in overweight and obesity so far when compared with healthy eating lean subjects, the cross-sectional nature of the study does not allow us to determine causality, and it is unclear whether taste dysfunction in obese patients is a consequence or a cause of abnormal nutritional and metabolic patterns. Further, for future perspectives—especially enlarging the study cohort—it could be of interest to deepen the interaction between single taste, biochemical regulators, routine blood samples, and nutrient intake. However, to better achieve such aims, attention should be paid to meal intervention and/or dietary habits and taste tests should be specifically devised in terms of tastant qualities and sensorial modalities [1]. Thus, given these assumptions, the present data have to be considered preliminary and should be replicated in further cohorts of patients. In particular, future studies may better highlight in cross-sectional and—especially—prospective manner those individual and environmental interactions that could better explain the causative connections of weight increase, food habits, and chemical senses changes.

## Figures and Tables

**Table 1:** Socio-demographic aspects and anthropometric and bioelectrical impedance analysis differences in lean, overweight, stage I, and stage II obesity participants.

	Lean (*n* = 60)	Overweight (*n* = 39)	Stage I Obesity (*n* = 18)	Stage II Obesity (*n* = 20)
Socio-demographic aspects				
Age (years)	54.04 ± 10.27	53.51 ± 11.17	54.3 ± 13.1	55 ± 11.81
Male	31	20	7	10
Female	29	19	11	10
Anthropometric and BIA measurements				
Waist circumference (cm)	86.99 ± 5.4 *#ƚ	97.46 ± 4.37 £$	105.72 ± 6.89 &	120.82 ± 12.55
Height (cm)	165.55 ± 11.38	168.28 ± 13.53	165.85 ± 9.62	166.91 ± 11.65
Weight (Kg)	69.16 ± 10.64 *#ƚ	79.38 ± 16.62 $	86.43 ± 10.76 &	106.77 ± 18.73
BMI (Kg/m^2^)	23.64 ± 1.47 *#ƚ	27.66 ± 1.4 £$	31.46 ± 1.38 &	38.08 ± 2.99
FM%	18.85 ± 5.17 *#ƚ	33.42 ± 4.13 £$	40.68 ± 6.99	45.23 ± 7.23
VFlevel	7.68 ± 2.69 *#ƚ	10.23 ± 2.54 £$	26.17 ± 4.16 &	24.17 ± 3.38
MM%	31.59 ± 4.58	28.16 ± 1.88 £$	12.61 ± 3.16	18.2 ± 5.38
REE (Kcal)	1509.15 ± 183.34	1574.1 ± 141.4 $	1650.94 ± 257.6 &	1915.65 ± 346.2

Socio-demographic aspects and main between-group effects in anthropometric and bioelectrical impedance analysis (BIA) measurements in lean, overweight, stage I, and stage II obesity participants. Body mass index: BMI; fat mass: FM; visceral fat level: VFlevel; muscle mass: MM; resting energy expenditure: REE; kilocalories: Kcal; centimeter: cm; meter: m; kilogram: Kg. Values are given in mean ± standard deviation. ∗, #, and ƚ, respectively, indicate significant (*p*-value < 0.008) comparisons between lean and overweight, lean and stage I obesity, and lean and stage II obesity participants. £, $, and &, respectively, indicate significant comparisons between overweight and stage I obesity, overweight and stage II obesity, and between stage I and stage II obesity participants.

**Table 2:** Nutritional aspects of lean, overweight, stage I, and stage II obesity participants.

	Lean (*n* = 31)	Overweight (*n* = 26)	Stage I Obesity (*n* = 11)	Stage II Obesity (*n* = 11)
Energy intake (kcal/day)	1897.37 ± 202.51 *#ƚ	3507.8 ± 603.67 £$	7108.58 ±1928.03	7133.42 ± 1584.5
Carbohydrate (g/day)	140.69 ± 27.43 *#ƚ	356.05 ± 139.2	485.82 ± 147.5	482.72 ± 176.51
Protein (g/day)	84.63 ± 18.06 *#ƚ	211.68 ± 59.46 £$	424.01 ± 138.63	460.55 ± 134.96
Fat (g/day)	100.41 ± 23.1 *#ƚ	189.96 ± 58.77 £$	297.25 ± 95.36	318.58 ± 63.57
Saturated fat (g/day)	26.27 ± 9.25 *#ƚ	82.2 ± 10.15 $	87.22 ± 33.24	108.62 ± 33.99
Monounsaturated fatty acids (g/day)	35.64 ± 8.19 *#ƚ	87.5 ± 24.12 £$	144.38 ± 55.12	158.1 ± 44.27
Polyunsaturated fatty acids (g/day)	6.19 ± 2.33 *#ƚ	17.07 ± 3.03 £$	28.46 ± 10.37	37.93 ± 29.6
n-3 Fatty acids (g/day)	1.01 ± 0.53 *#ƚ	4.16 ± 0.9 £	5.85 ± 2.71	19.92 ± 47.31
n-6 Fatty acids (g/day)	5.92 ± 1.9 *#ƚ	11.62 ± 2.35 £$	22.13 ± 8	37.05 ± 46.57
Cholesterol (mg/day)	232.55 ± 46.82 *#ƚ	836.09 ± 320.43 £$	1362.52 ± 606.19	1396.44 ± 395.68
Alcohol (g/day)	5.73 ± 3.48 *#ƚ	20.14 ± 8.49 £$	50.17 ± 54.59	53.13 ± 62.23
Fiber (g/day)	14.72 ± 5.29 *#ƚ	62.31 ± 16.16 £$	109.72 ± 35.65	108.2 ± 52.46
Sodium (mg/day)	1207.86 ± 379.23 *#ƚ	1838.93 ± 735.08 £$	6683.02 ± 4380.24	8456.19 ± 2803.17
Potassium (mg/day)	2253.88 ± 715.06 #ƚ	2706.15 ± 805.1 £$	14092.83 ± 3329.49	14069.59 ± 4337.26

Main between-group effects of nutritional status evaluated by means of the food frequency questionnaire in lean, overweight, stage I, and stage II obesity participants. Kilocalories: Kcal; gram: g; milligram: mg; microgram: µg. Values are given in mean ± standard deviation. ∗, #, and ƚ, respectively, indicate significant (*p*-value < 0.008) comparisons between lean and overweight, lean and stage I obesity, and lean and stage II obesity participants. £ and $, respectively, indicate significant comparisons between overweight and stage I obesity, overweight and stage II obesity, and between stage I and stage II obesity participants.

**Table 3:** Biochemical assay parameter differences in stage I and stage II obesity participants.

	Lean (*n* = 60)	Overweight (*n* = 39)	Stage I Obesity (*n* = 18)	Stage II Obesity (*n* = 20)
Total cholesterol (mg/dl)	167.58 ± 18.76 *#ƚ	188.41 ± 20.03 £$	216.27 ± 49.64	206.2 ± 28.95
LDL (mg/dl)	91.88 ± 22.71 #ƚ	100.68 ± 19.8 £$	135.43 ± 35.8	131.86 ± 33.76
HDL (mg/dl)	50.91 ± 8.8 *#	61.43 ± 5.68	62.22 ± 18.4	57.85 ± 21.42
Triglycerides (mg/dl)	123.9 ± 22.44 ƚ	131.43 ± 23.34	116 ± 70.56	153.8 ± 79.28
Serum glucose (mg/dl)	83.63 ± 8.94 *#ƚ	92.97 ± 9 $	96.44 ± 8.21 &	114.65 ± 22.35
Creatinine (mg/dl)	0.85 ± 0.19	0.88 ± 0.18	0.82 ± 0.15	0.88 ± 0.2
AST (U/L)	12.4 ± 3.42 *#ƚ	16.1 ± 4.29 £$	23.11 ± 6.29	23.83 ± 7.64
ALT (U/L)	13.3 ± 3.57 *#ƚ	16.84 ± 5.01 £$	23.5 ± 10.98	27.46 ± 15.02

Main between-group effects in biochemical assay parameters in lean, overweight, stage I, and stage II obesity participants. Low-density lipoprotein: LDL; high-density lipoprotein: HDL; aspartate transaminase: AST; alanine aminotransferase: ALT; milligram: mg; deciliter: dl; liter: L; international unit: U. Values are given in mean ± standard deviation. ∗, #, and ƚ, respectively, indicate significant (*p*-value < 0.008) comparisons between lean and overweight, lean and stage I obesity, and lean and stage II obesity participants. £, $, and &, respectively, indicate significant comparisons between overweight and stage I obesity, overweight and stage II obesity, and between stage I and stage II obesity participants.

**Table 4:** Multiple regression model of total taste scores in relation to socio-demographic and anthropometric factors, FFQ-based nutrient intakes, and biochemical assays in all the participants.

	Partial Regression Coefficient	Std. Err	t	*p*-Value	Cnf. Lmt−95.00%	Cnf. Lmt+95.00%	Partial Correlation Coefficient (ß)	Std. Err. ß	Cnf. Lmt−95.00%	Cnf. Lmt+95.00%
Socio-demographic and anthropometric factors										
Intercept	38.499	22.65	1.699	0.041	−6.332	83.331				
Age	−0.146	0.023	−6.153	<0.001	−0.193	−0.099	−0.453	0.073	−0.599	−0.307
VFlevel	−0.167	0.082	−2.038	0.043	−0.329	−0.004	−0.173	0.084	−0.341	−0.005
Gender	−1.335	0.413	−3.232	0.001	−2.153	−0.517	−0.201	0.062	−0.325	−0.078
FFQ-based nutrients intakes										
Intercept	26.111	0.717	36.37	<0.001	24.689	27.532				
Monounsaturated fatty acids	−0.013	0.005	−2.183	0.032	−0.024	−0.001	−0.203	0.093	−0.389	−0.017
Biochemical assays										
Intercept	33.139	4.113	8.056	<0.001	24.997	41.281				
Leptin	−0.201	0.06	−3.36	0.001	−0.32	−0.082	−0.367	0.109	−0.584	−0.151

Table depicting significant results of the stepwise regression model of total taste scores in relation to socio-demographic and anthropometric factors, FFQ-based nutrient intakes, and biochemical assays in all the participants. Std.: standard; Err.: error; Cnf.: confidence; Lmt.: limit; VFlevel: visceral fat level; food frequency questionnaire: FFQ.

## Data Availability

Not applicable.

## Supplementary Materials

The following supporting information can be downloaded at: https://www.mdpi.com/article/10.3390/nu15051114/s1, Table S1: Multiple regression model of total taste scores in relation to socio-demographic and anthropometric factors, FFQ-based nutrients intakes and biochemical assays in all the participants.
