# Supplementary material for: Taste Function in Adult Humans from Lean Condition to Stage II Obesity: Interactions with Biochemical Regulators, Dietary Habits, and Clinical Aspects
Source: Nutrients. 2023 Feb 23;15(5):1114. doi: 10.3390/nu15051114 (PMC10005537; doi:10.3390/nu15051114)
Supplement: Supplementary file 1 [file nutrients-15-01114-s001.zip › nutrients-2188480-supplementary.pdf]

Table S1: Multiple regression model of total taste scores in relation to socio-demographic and anthropometric factors, FFQ-based nutrients intakes and biochemical assays in all the participants.

	Partial regression coefficient	Std.Err	t	p-value	Cnf.Lmt -95,00%	Cnf.Lmt +95,00%	Partial correlation coefficient (ß)	Std.Err. ß	Cnf.Lmt -95,00%	Cnf.Lmt +95,00%
**Socio-demographic and anthropometric factors**										
**Intercept**	**38.499**	**22.65**	**1.699**	**0.041**	**-6.332**	**83.331**				
**age**	**-0.146**	**0.023**	**-6.153**	**< 0.001**	**-0.193**	**-0.099**	**-0.453**	**0.073**	**-0.599**	**-0.307**
**Waist circumference (cm)**	0.024	0.036	0.684	0.495	-0.047	0.096	0.071	0.104	-0.135	0.279
**Height (cm)**	-0.003	0.148	-0.021	0.983	-0.296	0.29	-0.01	0.518	-1.036	1.014
**Weight (Kg)**	0.092	0.156	0.591	0.555	-0.217	0.402	0.406	0.686	-0.953	1.765
**BMI (Kg/m^2)**	-0.398	0.444	-0.897	0.37	-1.278	0.48	-0.425	0.474	-1.364	0.512
**FM%**	-0.053	0.046	-1.151	0.251	-0.145	0.038	-0.174	0.151	-0.475	0.125
**MM%**	-0.078	0.073	-1.071	0.285	-0.224	0.066	-0.106	0.099	-0.302	0.089
**VFlevel**	**-0.167**	**0.082**	**-2.038**	**0.043**	**-0.329**	**-0.004**	**-0.173**	**0.084**	**-0.341**	**-0.005**
**REE (Kcal)**	0.001	0.001	1.437	0.153	0	0.004	0.102	0.071	-0.038	0.244

**gender**	**-1.335**	**0.413**	**-3.232**	**0.001**	**-2.153**	**-0.517**	**-0.201**	**0.062**	**-0.325**	**-0.078**
	**FFQ-based nutrients intakes**									
**Intercept**	**21.979**	**1.107**	**19.838**	**< 0.001**	**19.766**	**24.193**				
**Energy intake (kcal/day)**	0	0	-0.118	0.906	-0.001	0.001	-0.051	0.432	-0.914	0.812
**Carbohydrate (g/day)**	0.001	0.002	0.492	0.624	-0.004	0.006	0.073	0.149	-0.224	0.371
**Protein (g/day)**	0.005	0.006	0.906	0.367	-0.006	0.017	0.255	0.282	-0.307	0.819
**Fat (g/day)**	-0.001	0.006	-0.238	0.811	-0.014	0.011	-0.044	0.185	-0.414	0.325
**Saturated fat (g/day)**	0.032	0.018	1.75	0.084	-0.004	0.068	0.34	0.194	-0.048	0.728
**Monounsaturated fatty acids (g/day)**	**-0.013**	**0.005**	**-2.183**	**0.032**	**-0.024**	**-0.001**	**-0.203**	**0.093**	**-0.389**	**-0.017**
**Polyunsaturated fatty acids (g/day)**	0.095	0.086	1.098	0.276	-0.078	0.268	0.434	0.396	-0.356	1.226
**n-3 Fatty acids (g/day)**	0.032	0.122	0.268	0.789	-0.211	0.276	0.168	0.629	-1.088	1.426
**n-6 Fatty acids (g/day)**	-0.102	0.122	-0.838	0.404	-0.348	0.142	-0.587	0.7	-1.987	0.811
**Cholesterol (mg/day)**	0	0.001	0.042	0.966	-0.002	0.002	0.007	0.169	-0.33	0.345
**Alcohol (g/day)**	-0.007	0.012	-0.611	0.542	-0.033	0.017	-0.087	0.142	-0.372	0.197
**Fiber (g/day)**	0.007	0.014	0.529	0.598	-0.02	0.035	0.097	0.183	-0.269	0.463
**Sodium (mg/day)**	0	0	-1.243	0.218	0	0	-0.256	0.206	-0.669	0.155
**Potassium (mg/day)**	0	0	1.206	0,232	0	0	0,288	0,239	-0,189	0,767

	Biochemical assays									
**Intercept**	**33.139**	**4.113**	**8.056**	**< 0.001**	**24.997**	**41.281**				
**Leptin (ng/ml)**	-0.201	0.06	-3.36	0.001	-0.32	-0.082	-0.367	0.109	-0.584	-0.151
**Ghrelin (pg/ml)**	-0.002	0.002	-1.062	0.29	-0.007	0.002	-0.147	0.138	-0.421	0.127
**Total cholesterol (mg/dl)**	-0.016	0.066	-0.243	0.808	-0.148	0.115	-0.176	0.725	-1.611	1.258
**LDL (mg/dl)**	0.007	0.065	0.109	0.912	-0.122	0.136	0.068	0.623	-1.166	1.302
**HDL (mg/dl)**	-0.008	0.071	-0.121	0.903	-0.15	0.133	-0.033	0.272	-0.573	0.506
**Triglycerides (mg/dl)**	0.012	0.015	0.76	0.448	-0.019	0.043	0.149	0.196	-0.239	0.538
**Serum glucose (mg/dl)**	-0.017	0.03	-0.575	0.565	-0.077	0.042	-0.054	0.094	-0.242	0.132
**Insulin (μU)**	-0.007	0.083	-0.087	0.93	-0.172	0.157	-0.007	0.083	-0.172	0.158
**AST (U/L)**	0.003	0.068	0.045	0.963	-0.132	0.138	0.005	0.13	-0.251	0.263
**ALT (U/L)**	-0.091	0.05	-1.813	0.072	-0.191	0.008	-0.215	0.118	-0.45	0.019
**Creatinine (mg/dl)**	0.68	1.526	0.445	0.656	-2.34	3.701	0.036	0.082	-0.126	0.2

Table S1. Table depicting significant and not significant results of the stepwise regression model of total taste scores in relation to socio-demographic and anthropometric factors, FFQ-based nutrients intakes and biochemical assays in all the participants. Std., standard; Err., error; Cnf., confidence; Lmt., limit; VFlevel, visceral fat level; food frequency questionnaire, FFQ. In bold significant (< 0.05) differences. For specific acronyms please refer to Figures and Tables included in the text.
